# Characterization of Phase I Hepatic Metabolites of Anti-Premature Ejaculation Drug Dapoxetine by UHPLC-ESI-Q-TOF

**DOI:** 10.3390/molecules26133794

**Published:** 2021-06-22

**Authors:** Robert Skibiński, Jakub Trawiński, Maciej Gawlik

**Affiliations:** Department of Medicinal Chemistry, Faculty of Pharmacy, Medical University of Lublin, Jaczewskiego 4, 20-090 Lublin, Poland; jakub.trawinski@umlub.pl (J.T.); maciej.gawlik@umlub.pl (M.G.)

**Keywords:** mass spectrometry, chromatography, metabolites, biotransformation, HLM, in silico toxicity

## Abstract

Determination of the metabolism pathway of xenobiotics undergoing the hepatic pass is a crucial aspect in drug development since the presence of toxic biotransformation products may result in significant side effects during the therapy. In this study, the complete hepatic metabolism pathway of dapoxetine established according to the human liver microsome assay with the use of a high-resolution LC–MS system was described. Eleven biotransformation products of dapoxetine, including eight metabolites not reported in the literature so far, were detected and identified. *N*-dealkylation, hydroxylation, *N*-oxidation and dearylation were found to be the main metabolic reactions for the investigated xenobiotic. In silico analysis of toxicity revealed that the reaction of didesmethylation may contribute to the increased carcinogenic potential of dapoxetine metabolites. On the other hand, *N*-oxidation and aromatic hydroxylation biotransformation reactions possibly lead to the formation of mutagenic compounds.

## 1. Introduction

Xenobiotics are substances that are foreign to human organism, and therefore they are subjected to undergo various biochemical reactions. Drug metabolism is a complex process divided into two phases. In general, it aims at changing the chemical character of the molecule to more hydrophilic so as to facilitate elimination of biotransformation products, mainly in urine and feces. Phase I reactions including dealkylation as well as the introduction of oxygen into the molecule structure in the course of such reactions as hydroxylation or *N*-oxidation constitute the first step of the biotransformation process of xenobiotics. That may be followed by the conjugation of formed intermediates with endogenic cofactors, essentially glucuronic or sulfuric acid and glutathione, which constitutes phase II preceding the excretion of metabolites. These redox reactions, to a major extent, are catalyzed by the P450 microsomal superfamily of isoenzymes with its greatest presence in the tissues of the liver making it the principal site of drug metabolism [1,2,3,4,5]. Due to this reason, the liver tissue is mainly exposed to damage, which may cause severe impact on the patient’s health [6]. The understanding of the metabolism’s molecular basis as well as identification of the responsible organs resulted in the development of an in vitro metabolism simulation method assuming the use of human liver microsomes (HLM) as a frequently used protocol in this type of research [7,8]. Drug metabolism studies provide information about the formed metabolites allowing for their further assessments. The continuous progress in the analytical techniques support this process, allowing for the detection of the metabolites present only in trace levels in plasma. The use of a high-resolution mass spectrometer with quadrupole time-of-flight detection combined with liquid chromatography and a soft electrospray ionization source (LC–ESI-Q-TOF) is considered to be the gold standard in this type of research owing to its great sensitivity and efficiency [9,10,11,12,13,14,15].

Premature ejaculation (PE) is one of the most common male sexual disorders with prevalence in the population estimated in the range of 20–30% [16,17]. The understanding of the disease’s pathomechanism guided the development of dapoxetine which was originally dedicated for analgesia and depression treatment [18]. Until its approval, the treatment of PE was primarily conducted by the off-label use of selective serotonin reuptake inhibitor (SSRI) agents since an ejaculation delay is one of the most common side effects of this group of medicines. Depression treatment was the main concern of their use as they reached the therapeutic concentration in plasma after long-term administration. Dapoxetine stood out against this background due to having a more favorable pharmacokinetic profile regarding a short half-life and a rapid therapeutic effect, which constituted the foundation of its involvement in the PE treatment as a drug used as needed [19]. Dapoxetine similar to other SSRIs is extensively metabolized by the liver. At the same time, dapoxetine is meant to be a potential inhibitor of cytochrome P450 isoforms like other SSRIs and that possibility underlay this study’s aim to identify the complete hepatic biotransformation pathway of dapoxetine. It was reported that cytochrome P450 enzymes (CYP3A4 and CYP2D6) and flavin-containing monooxygenase 1 (FMO1) are mainly involved in dapoxetine metabolism forming *N*-oxide, *N*-desmethyldapoxetine and *N*,*N*-didesmethyldapoxetine as the major metabolites [19,20,21,22]. Moreover, structural similarity of the dapoxetine molecule to duloxetine suggested a possible similarity in the formed metabolites, especially in terms of the hydroxy ones [23].

In this study, we present the complete hepatic metabolism pathway of dapoxetine established according to the HLM assay with the use of a combined LC–MS system. In addition, toxicity prediction of the characterized metabolites was calculated with the use of several available in silico tools.

## 2. Results and Discussion

### 2.1. Metabolite Identification

**Figure 1:**
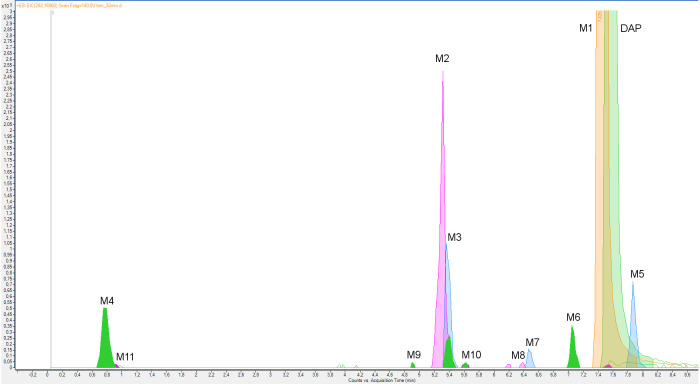
Overlapped extracted ion chromatograms (EIC) of dapoxetine (DAP) and its metabolites (M1–M11) after a 30-min incubation with HLM.

Eleven metabolites of dapoxetine were identified with the use of the MS/MS fragmentation spectra registered by a high-resolution LC-ESI-Q-TOF mass spectrometer (Figure 1). Fragmentation patterns of dapoxetine and its metabolites are presented in Table 1. Fragmentation of dapoxetine began with the loss of a dimethylamine fragment and formation of the *m/z* 261.1287 ion, which was also the most abundant in the whole MS/MS spectrum (Figure S1) [23]. Then, the gradual degradation of an alkoxy chain resulted in the formation of the *m/z* 183.0809, 157.0649, 145.638 and 129.0697 ions. Two fragments represented by the *m*/*z* 233.0967 and 215.0877 ions were formed as a result of the molecule rearrangement. The three low-mass fragments—*m*/*z* 117.0698, 91.0544 and 77.0369 ions—represented a phenylpropyl fragment. Such fragmentation pattern is typical for several metabolites, especially for the products of dapoxetine dealkylation—M1 and M6 (Figures S2 and S7, respectively). A similar MS/MS spectrum was also observed in the case of dapoxetine *N*-oxide (M5). The only distinction could be spotted in the low-mass region of the spectrum, where the *m*/*z* 74.0979 and 62.0610 ions (representing *N*-ethyl-*N*,*N*-dimethylamine and degraded *N*-oxide fragments) were present (Figure S6). The main difference observed between the fragmentation patterns of dapoxetine and its hydroxylated metabolites was the presence of 16-Da heavier ion series in the MS/MS spectra. For instance, in the case of M2 (Figure S3), the *m*/*z* 277.1220, 249.0909, 231.0830, 199.0748, 173.0597 and 159.0451 ions were observed instead of their nonhydroxylated analogs. Such pattern indicates that the additional hydroxyl group was placed in a naphthalene ring. Although its accurate location was not possible on the basis of MS/MS fragmentation, in the case of the most abundant hydroxylated metabolites, the four-position was suggested as probably the most preferred. This assumption was also supported by the work of Lantz et al. [24] who studied metabolic pathways of duloxetine which possesses an identical fragment in the structure. The low-mass region of the spectrum remained practically unchanged. MS/MS fragmentation patterns of the remaining metabolites possessing the 4-hydroxyl group (M3, M9 and M10—Figures S4, S10 and S11) were almost the same; however, in some cases, several low-abundant ions were not observed. Nevertheless, in the case of M10, the presence of the *m*/*z* 62.0615 ion indicated that the additional oxygen was attached to the *N*,*N*-dimethylamine fragment, forming an *N*-oxide derivative. Fragmentation patterns of the other hydroxylated metabolites (M7 and M8) also included the 16-Da heavier analogs of the ions present in the MS/MS spectrum of the parent compound (*m*/*z* 199.0774 and 145.0640 ions for M7—Figure S8; *m*/*z* 199.0769 and 145.0634 ions for M8—Figure S9), which similarly indicated hydroxylation of the naphthalene ring (at the same time, the presence of the *m*/*z* 117.0704 ion in both cases indicated that the additional hydroxyl group was not attached to the phenyl ring). Furthermore, in these cases, the most probable location of an additional OH group was suggested on the basis of the previously cited work—the second naphthalene ring was chosen (presumably five- or six-position [24]). A completely different MS/MS fragmentation pattern was observed in the case of M4 which was formed as a result of dapoxetine dearylation. The majority of the detected ions were low-mass fragments, observed also in the case of the parent compound (*m*/*z* 117.0689, 105.0698 and 91.0546). The only difference was the presence of the *m*/*z* 135.0797 ion which represented a 3-phenylpropanol fragment. M4 was probably formed as a result of the reduction of another metabolite—M11—which was an aldehyde as well as a primary product of the parent compound dearylation. The MS/MS fragmentation pattern of this metabolite (spectrum shown in Figure S12) was very similar to that observed in the case of M4. The only difference was the presence of the *m*/*z* 136.9301 ion (a product of acetaldehyde elimination) instead of the *m*/*z* 135.0797 ion.

### 2.2. HLM Biotransformation of Dapoxetine

**Figure 2:**
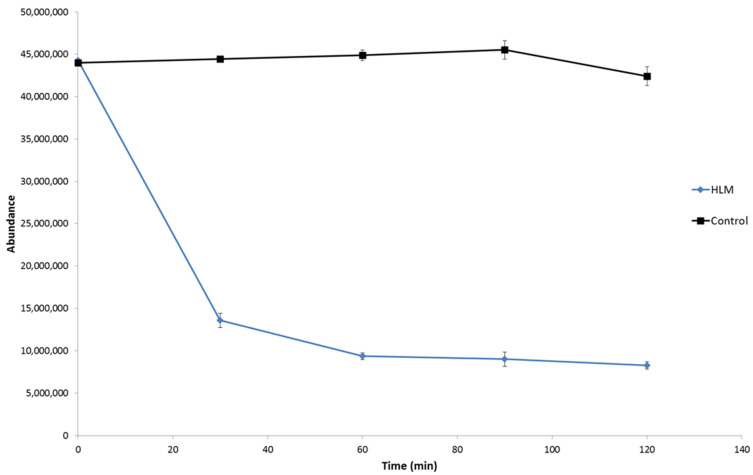
Biotransformation of dapoxetine in HLM (presented as the function of time; error bars represent the standard deviation of the mean, *n* = 3).

**Figure 3:**
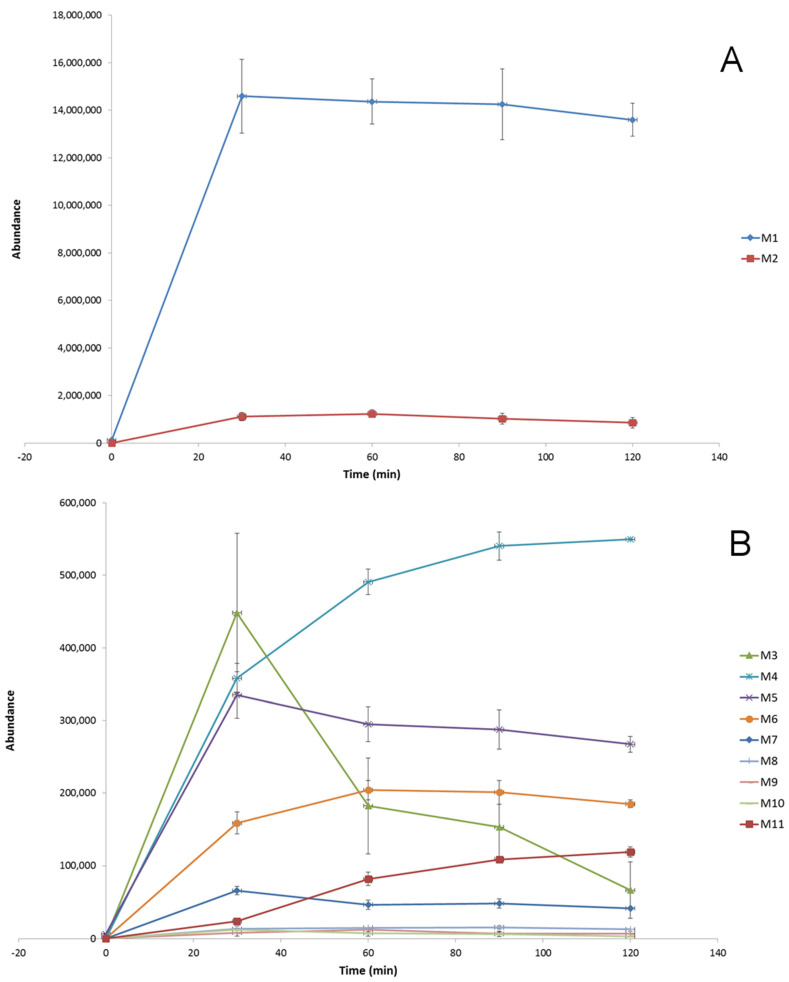
Evolution profiles of the dapoxetine metabolites (**A**,**B**) formed in HLM (presented as the function of time; error bars represent the standard deviation of the mean, *n* = 3).

The biotransformation kinetics of dapoxetine in HLM was investigated and, based on the evaluation of the abundance of the parent ion (*m*/*z* 306.1852), in the studied time range of incubation (0–120 min), rapid metabolism of the analyzed drug was observed (Figure 2). The obtained results showed that during 30 min of the hepatic microsome incubation, about 70% of dapoxetine was metabolized and, subsequently, the biotransformation process significantly slowed down. A similar observation was obtained for the evolution profiles for the ten ions formed during the in vitro incubation of dapoxetine with the isolated microsomes (Figure 3). The optimal metabolic reaction time for these ions was between 30- and 60-min incubation and afterwards their concentration decreased. The selected ten ions as the potential metabolites of dapoxetine were subjected to MS/MS analyses in order to perform their structural identification.

### 2.3. Hepatic Biotransformation Pathways

On the basis of the obtained results, it was observed that dealkylation is one of the most important metabolic reactions for the hepatic pass of dapoxetine. It is well-known that this drug is extensively metabolized by the liver and the CYP3A4 and CYP2D6 enzymes as well as FMO1 play the crucial role in this process, which results in *N*-oxidation and demethylation of dapoxetine [25]. In the following research, the main metabolite (M1) was an *N*-dealkylation product which was registered on a significantly higher level than the other metabolites (Figure 2). However, the other metabolic reactions such as hydroxylation, *N*-oxidation and dearylation also played a very important role in the formation of the majority of dapoxetine metabolites, i.e., the second most abundant metabolite (M2) was an effect of the combination of dealkylation and hydroxylation and for the other minor metabolites, hydroxylation was the crucial reaction (Table 1).

It should be noticed that the only three identified biotransformation products (M1, M5 and M6) are well-known metabolites of dapoxetine, but the rest of the identified metabolites are new and have not been described in the literature so far.

Noteworthily, hydroxylated metabolites are usually active and therefore their influence on the final pharmacological effect of the used pharmaceutical should be taken into account. Moreover, such kind of metabolites may exhibit improved pharmacokinetic behavior as compared to its parent drug [26].

**Figure 4:**
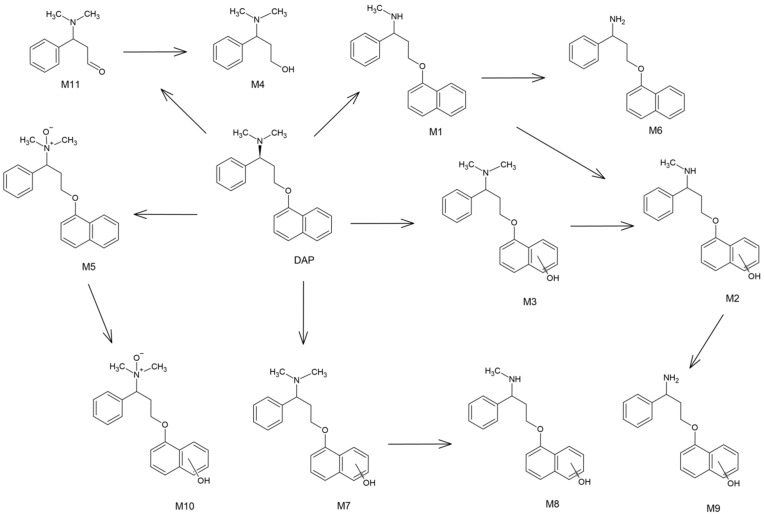
The proposed hepatic metabolic pathway of dapoxetine (location of the hydroxyl group involves the whole naphthalene ring).

The proposed complete phase I hepatic metabolic pathway of dapoxetine is presented in Figure 4.

### 2.4. In Silico Assessment of Toxicity

Although the use of in vivo or in vitro toxicity estimation methods is the most advisable, they are often expensive and time-consuming. On the other hand, according to numerous studies, the in silico methods based on the molecular descriptors can possess a sufficient prediction ability and may be a reasonable alternative for preliminary assessment of a chemical’s toxicity [25,27,28].

Mutagenicity (expressed as a probability of the positive outcome of the Ames test) and carcinogenicity were evaluated using the T.E.S.T., Percepta and Vega software (see detailed information concerning the applied models in Section S1 of the Supplementary Materials). In the case of both toxicity categories, dapoxetine was predicted as a nontoxic compound (see raw data in Table S2).

Although half of the detected metabolites were defined by the T.E.S.T. model as slightly mutagenic, only few relationships between the structure of the compounds and the outcome can be noticed. Firstly, the presence of the *N*-oxide group probably increases the mutagenic potential (positive results obtained for M5 and M10). The second noteworthy finding is a correlation between the predicted mutagenicity and the presence of both the hydroxylated naphthalene ring and the *N*,*N*-dimethylamine group (M3 and M7). Besides the aforementioned compounds, positive prediction was also obtained for M9. On the other hand, according to the Percepta model, neither dapoxetine nor its metabolites were defined as mutagenic. Moreover, all of the studied biotransformation products possessed a lower mutagenic potential than the parent compound. Mutagenicity predicted for M2–M4, M6, M9 and M11 was exceptionally low (below 0.2), which only partially squares with the outcomes given by the T.E.S.T. model.

In the case of the carcinogenic potential, notable is the positive correlation of outcomes and presence of the primary amine group (M6 and M9). Although the applied model also predicted carcinogenic properties for M5, the other metabolite possessing the *N*-oxide group (M10) was not defined as a carcinogen. The toxic potential in this class was also predicted for the product of dapoxetine dearylation (M4).

Taking into consideration the discussed toxicity estimation outcomes as well as the relative amounts of the detected metabolites, it should be noted that the potentially harmful properties were predicted for the minor biotransformation products. Therefore, their contribution to the resultant toxic properties of the formed metabolites mixture may not be significant under the in vivo conditions.

## 3. Materials and Methods

### 3.1. Chemicals and Reagents

Dapoxetine hydrochloride ((S)-*N*,*N*-dimethyl-1-phenyl-3-(1-naphthalenyloxy)propanamine hydrochloride, purity ≥ 98%), water (LC–MS grade), β-nicotinamide adenine dinucleotide 2′-phosphate-reduced tetrasodium salt hydrate (NADPH), HLM, sodium phosphate monobasic monohydrate salt and sodium phosphate dibasic anhydrous salt were obtained from Sigma-Aldrich (St. Louis, CA, USA). Acetonitrile (hypergrade for LC–MS) was purchased from Merck (Darmstadt, Germany) and 98% formic acid (MS grade) was obtained from Fluka (Taufkirchen, Germany).

### 3.2. In Vitro Simulation of Metabolism by HLM

Metabolism reactions were performed in vitro with the use of the human liver microsome fraction. The incubation system consisted of 50 μM substrate, 55 mM phosphate buffer (pH 7.4) and 0.5 mg·mL^−1^ HLM. Following the 2-min preincubation period at 37 °C, metabolic reactions were initiated by the addition of 10 μL NADPH (20 mM). The total volume of the reaction suspension was equal to 200 μL. The reaction was terminated after 0, 30, 60, 90 and 120 min of incubation with the use of 200 μL ice-cold acetonitrile–methanol mixture (1:1). Next, the precipitated samples were centrifuged at 15,000 rpm for 10 min at 4 °C and the supernatants (40 μL) were transferred into the vials for the LC–MS analysis. The negative control samples were prepared in the same manner without adding the NADPH solution.

### 3.3. Analytical Procedures

The LC–MS analysis was performed with the use of an Agilent 6520 series high-resolution Q-TOF system and a UHPLC 1290 series system (Agilent Technologies, Santa Clara, CA, USA) with a Kinetex C18 (2.1 × 50 mm, dp = 1.7 μm) column and a C18 precolumn guard (Phenomenex, Torrance, USA). In order to perform both qualitative and quantitative analysis of the studied processes, the MS detector was operated in the positive mode in the extended dynamic range (2 GHz). MassHunter workstation software version B.04.00 was used for the control of the system, data acquisition, qualitative and quantitative analysis. To ensure accuracy in mass measurements, a reference mass correction was applied and masses 121.050873 and 922.009798 were used as lock masses. MS detection based on the extracted ion current chromatograms (EIC) was applied for the quantitative analysis of dapoxetine and its metabolites; next, the automatic MS/MS mode (using the abundance algorithm) was used to register their fragmentation spectra. All the chromatographic and spectrometric parameters are described in Table S1.

### 3.4. In Silico Assessment of the Toxicity of Dapoxetine and Its Metabolites

In silico toxicity including mutagenicity (expressed as the probability of the positive outcome of the Ames test) and carcinogenicity (calculated using the CAESAR 2.1.9 model) were evaluated using the Toxicity Estimation Software Tool (T.E.S.T.) v. 4.2.1, ACD/Percepta 14.0.0 (ACD/Labs, 2015 Release) and Vega v. 1.1.4 software.

## 4. Conclusions

As the main purpose of this research, eleven hepatic biotransformation products of dapoxetine were detected and structurally characterized with the use of a combined high-resolution LC–MS system. Eight of the identified metabolites had not been reported in the literature thus far. Moreover, *N*-dealkylation, hydroxylation, *N*-oxidation and dearylation were found to be the main metabolic reactions for the investigated drug. The major metabolite was found to be a well-known desmethylation product of dapoxetine. Interestingly, in this study, we found that this metabolic reaction progressed even further via a previously not described hydroxylation pathway. What is more, the aforementioned product (*N*-desmethyl-4-hydroxydapoxetine) was the second most abundant metabolite of the studied substance. The in silico toxicity study showed that the presence of the *N*-oxide group as well as the presence of both the hydroxylated naphthalene ring and the *N*,*N*-dimethylamine group may increase (although not significantly) the mutagenic potential of dapoxetine metabolites. Nevertheless, even in the case of mutagenic (positive) compounds, the obtained values only slightly exceeded the critical 0.5 level. Such observations in connection with the lack of positive outcomes predicted by the second model suggest that, in fact, mutagenicity of dapoxetine metabolites is rather unlikely. In the case of carcinogenicity, it was observed that the toxic potential was elevated in the case of the didesmethylated biotransformation products of dapoxetine.

## Figures and Tables

**Table 1:** Q-TOF accurate mass elemental composition and MS/MS fragmentation of the analyzed metabolites.

Name	Reaction Type	Retention TIMe (min)	Measured Mass (*m*/*z*)	Theoretical Mass (*m*/*z*)	Mass Error (ppm)	Molecular Formula ([M + H]^+^)	MS/MS Fragmentation (*m*/*z*) and Formulas ([M + H]^+^)
DAP	–	7.55	306.1843	306.1852	−2.94	C_21_H_24_NO	261.1287 (C_19_H_17_O); 233.0967 (C_17_H_13_O); 215.0877 (C_17_H_11_); 183.0809 (C_13_H_11_O); 157.0649 (C_11_H_9_O); 145.0638 (C_10_H_9_O); 129.0697 (C_10_H_7_); 117.0698 (C_9_H_9_); 91.0544 (C_7_H_7_); 77.0396 (C_6_H_5_)
M1	Dealk	7.43	292.1687	292.1696	−3.08	C_20_H_22_NO	261.1272 (C_19_H_17_O); 243.1159 (C_19_H_15_); 233.0955 (C_17_H_13_O); 215.0852 (C_17_H_11_); 183.0804 (C_13_H_11_O); 157.0645 (C_11_H_9_O); 145.0638 (C_10_H_9_O); 129.0698 (C_10_H_7_); 117.0701 (C_9_H_9_); 101.0384 (C_8_H_5_); 91.0549 (C_7_H_7_); 77.0392 (C_6_H_5_)
M2	Dealk Ar-OH	5.31	308.1634	308.1645	−3.57	C_20_H_22_NO_2_	277.1220 (C_19_H_17_O_2_); 249.0909 (C_17_H_13_O_2_); 231.0830 (C_17_H_11_O); 199.0748 (C_13_H_11_O_2_); 173.0597 (C_11_H_9_O_2_); 159.0451 (C_10_H_9_O_2_); 145.0655 (C_10_H_9_O); 127.0545 (C_10_H_7_); 117.0696 (C_9_H_9_); 105.0703 (C_8_H_9_); 91.0548 (C_7_H_7_)
M3	Ar-OH	5.36	322.1770	322.1801	−9.62	C_21_H_24_NO_2_	277.1277 (C_19_H_17_O_2_); 249.0906 (C_17_H_13_O_2_); 199.0749 (C_13_H_11_O_2_); 173.0593 (C_11_H_9_O_2_); 145.0649 (C_10_H_9_O); 117.0702 (C_9_H_9_)
M4	Dearyl Red	0.75	180.1373	180.1383	−5.55	C_11_H_18_NO	135.0797 (C_9_H_11_O); 117.0689 (C_9_H_9_); 105.0698 (C_8_H_9_); 91.0546 (C_7_H_7_)
M5	*N*-ox	7.86	322.1770	322.1801	−9.62	C_21_H_24_NO_2_	261.1271 (C_19_H_17_O); 233.0940 (C_17_H_13_O); 183.0802 (C_13_H_11_O); 157.0638 (C_11_H_9_O); 129.0694 (C_10_H_7_); 117.0698 (C_9_H_9_); 74.0979 (C_4_H_12_N); 62.0610 (C_2_H_8_NO)
M6	Dealk	7.04	278.1528	278.1539	−3.95	C_19_H_20_NO	261.1263 (C_19_H_17_O); 233.0973 (C_17_H_13_O); 183.0804 (C_13_H_11_O); 157.0646 (C_11_H_9_O); 129.0687 (C_10_H_7_); 117.0690 (C_9_H_9_)
M7	Ar-OH	6.46	322.1770	322.1801	−9.62	C_21_H_24_NO_2_	277.1218 (C_19_H_17_O_2_); 199.0774 (C_13_H_11_O_2_); 145.0640 (C_10_H_9_O); 117.0704 (C_9_H_9_)
M8	Dealk Ar-OH	6.39	308.1634	308.1645	−3.57	C_20_H_22_NO_2_	277.1231 (C_19_H_17_O_2_); 145.0640 (C_10_H_9_O); 117.0703 (C_9_H_9_)
M9	Dealk Ar-OH	4.90	294.1479	294.1489	−3.40	C_19_H_20_NO_2_	277.1225 (C_19_H_17_O_2_); 199.0744 (C_13_H_11_O_2_); 173.0583 (C_11_H_9_O_2_); 177.0699 (C_9_H_9_)
M10	*N*-ox Ar-OH	5.62	338.1724	338.1751	−7.98	C_21_H_24_NO_3_	277.1218 (C_19_H_17_O_2_); 199.0737 (C_13_H_11_O_2_); 173.0600 (C_11_H_9_O_2_); 117.0698 (C_9_H_9_); 62.0615 (C_2_H_8_NO)
M11	Dearyl	0.88	178.1216	178.1226	−5.61	C_11_H_16_NO	136.9301 (C_9_H_14_N); 117.0709 (C_9_H_9_); 105.0710 (C_8_H_9_); 91.0551 (C_7_H_7_)

Dealk—dealkylation; Dearyl—dearylation; Ar-OH—aryl hydroxylation; *N*-ox—*N*-oxidation; Red—reduction.

## Data Availability

Data is contained within the article or Supplementary Materials.

## Supplementary Materials

The following are available online, Table S1: Applied LC and MS/MS parameter; Table S2: Predicted toxic properties of dapoxetine and its metabolites; Figure S1: MS/MS spectrum and fragmentation pattern of dapoxetine; Figure S2: MS/MS spectrum and fragmentation pattern of M1; Figure S3: MS/MS spectrum and fragmentation pattern of M2; Figure S4: MS/MS spectrum and fragmentation pattern of M3; Figure S5: MS/MS spectrum and fragmentation pattern of M4; Figure S6. MS/MS spectrum and fragmentation pattern of M5; Figure S7: MS/MS spectrum and fragmentation pattern of M6; Figure S8: MS/MS spectrum and fragmentation pattern of M7; Figure S9: MS/MS spectrum and fragmentation pattern of M8; Figure S10: MS/MS spectrum and fragmentation pattern of M9; Figure S11: MS/MS spectrum and fragmentation pattern of M10; Figure S12: MS/MS spectrum and fragmentation pattern of M11

## Sample Availability

Samples of the compounds are not available.
