# Supplementary material for: Characterization of Phase I Hepatic Metabolites of Anti-Premature Ejaculation Drug Dapoxetine by UHPLC-ESI-Q-TOF
Source: Molecules. 2021 Jun 22;26(13):3794. doi: 10.3390/molecules26133794 (PMC8270242; doi:10.3390/molecules26133794)
Supplement: Supplementary file 1 [file molecules-26-03794-s001.zip › molecules-1220719-supplementary.pdf]

Supplementary material

Characterization of Phase I Hepatic Metabolites of Anti Premature Ejaculation Drug - Dapoxetine by UHPLC-ESI-Q-TOF

Robert Skibiński*, Jakub Trawiński, Maciej Gawlik

Department of Medicinal Chemistry, Faculty of Pharmacy, Medical University of Lublin, Jaczewskiego 4, 20-090 Lublin, Poland

* Corresponding author. Tel.: +48 81 4487390; fax: +48 81 4487380.

E-mail address: robert.skibinski@umlub.pl

Secction S1.

Ames test model provided by ACD/Labs Percepta software is based on the database containing information about 5511 chemicals, whose mutagenic properties were tested experimentally. Those compounds were divided into the following groups, taking into account the outcomes of the Ames tests: positive, negative, weakly positive and inconclusive. All the records contain also information about: used bacteria strain (TA97, TA98, TA100, TA102, TA104, TA1535, TA1537, TA1538, E. Coli), potential metabolic activation, tested dose range, used solvents and method of experiment.

In the case of T.E.S.T. mutagenicity model, a database consisting of 5743 compounds, which were experimentally tested for potential of inducing the frame-shift mutations or base-pair substitutions in the histidine-dependent strains of Salmonella typhimurium, was applied. In fact, the software calculates the mutagenic potential of the unknown compound using three QSAR models: hierarchical clustering (HC), nearest neighbor (NN) and FDA model. Then the Consensus model (which was used in our study) is built by simply averaging of the three obtained outcomes. Due to different applicability domains of the HC, NN and FDA models, the Consensus model is able to provide the highest coverage and the best accuracy of prediction.

Carcinogenic potential was calculated using CAESAR model version 2.1.9 (Vega software). In this case the QSAR classification model was based on the neural network (NN) approach. The obtained outcomes are assigned to one of the following classes: Positive or Non-Positive (compound is assigned to the class having value greater than 0.5).

Table S1. Applied LC and MS/MS parameter

Device	Parameter	Value
LC	Solvents	A – 0.1% aqueous solution of HCOOH
		B – acetonitrile
	Gradient	10% B to 40% B
	Analysis time	9 min
	Post-time equilibration	2 min
	Flow rate	0.3 mL min^{-1}
	Injection volume	2 µL
	Column temperature	35 °C
MS	Ion source	Electrospray (ESI)
	Mode	Positive
	Source temperature	300 °C
	Drying gas flow	10 L min^{-1}
	Nebulizer pressure	40 psig
	Capillary voltage	3500 V
	Fragmentor voltage	140 V
	Skimmer voltage	65 V
	Octopole voltage	750 V
	Mass range	90 – 950 m/z
	Acquisition rate	1.5 spectra s^{-1}

Table S2. Predicted toxic properties of dapoxetine and its metabolites

Commpound	Mutagenicity Percepta	Mutagenicity T.E.S.T.	Carcinogenicity
DAP	0.28	0.44	–
M1	0.24	0.37	–
M2	0.14	0.47	–
M3	0.13	0.51	–
M4	0.09	0.14	+
M5	0.25	0.52	+
M6	0.16	0.26	+
M7	0.22	0.52	–
M8	0.25	0.24	–
M9	0.15	0.61	+
M10	0.25	0.59	–
M11	0.19	0.56	–

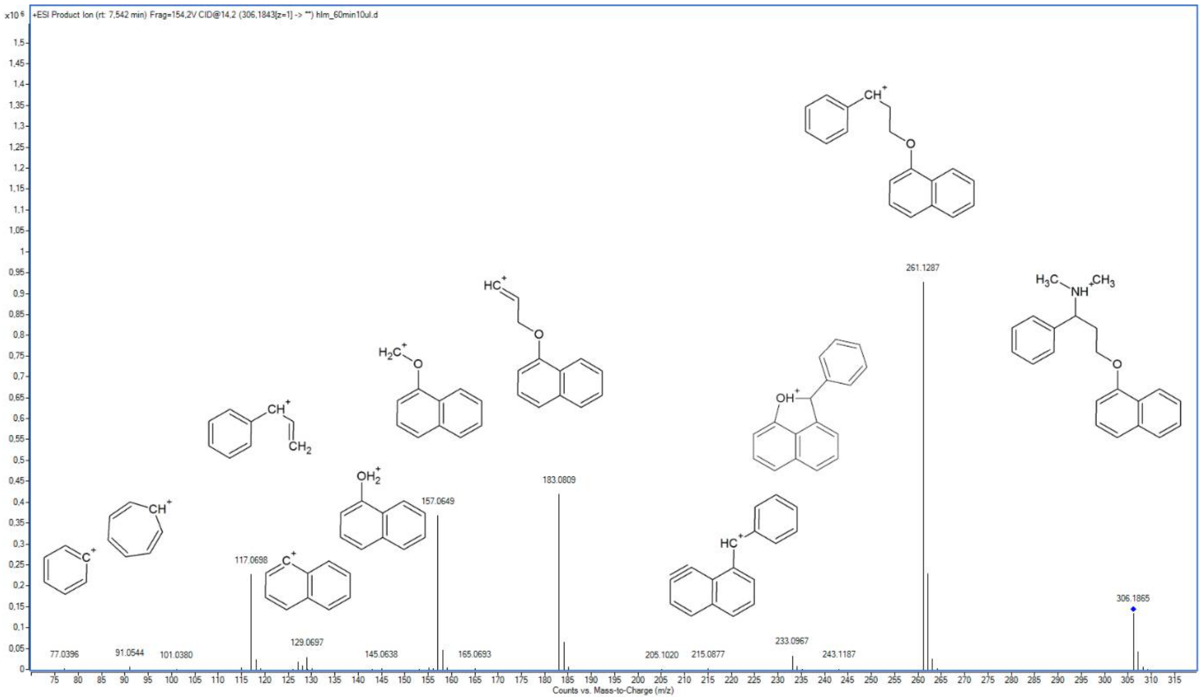

Figure S1. MS/MS spectrum and fragmentation pattern of dapoxetine

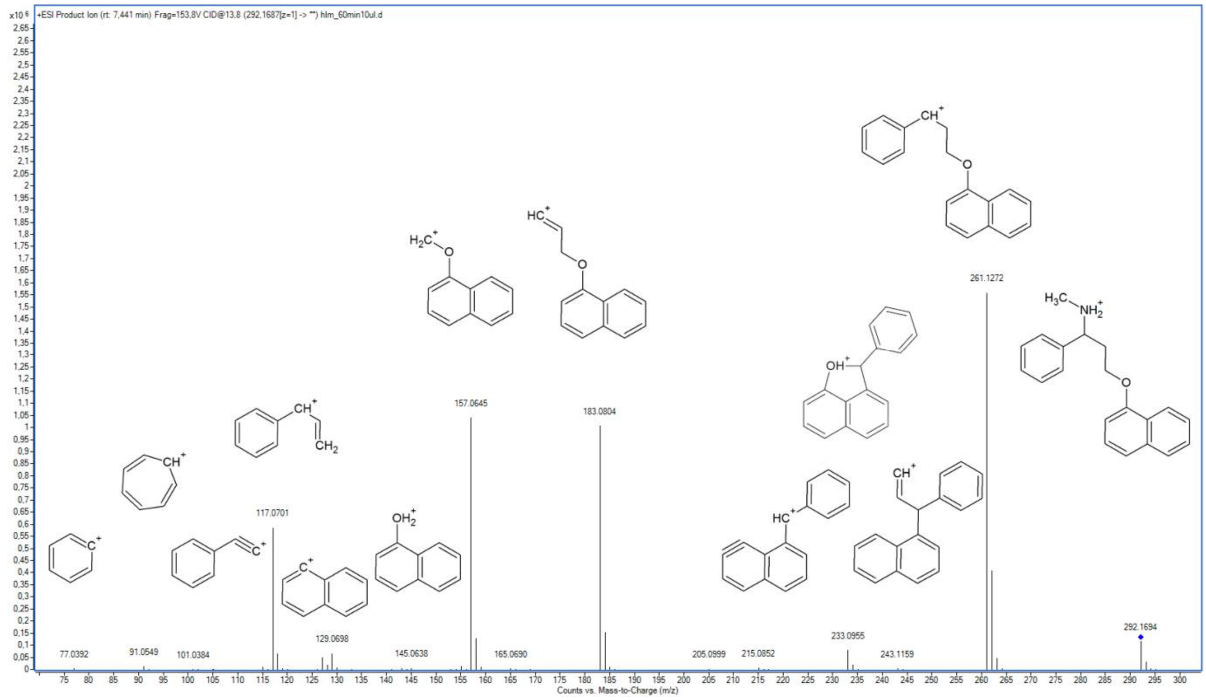

Figure S2. MS/MS spectrum and fragmentation pattern of M1

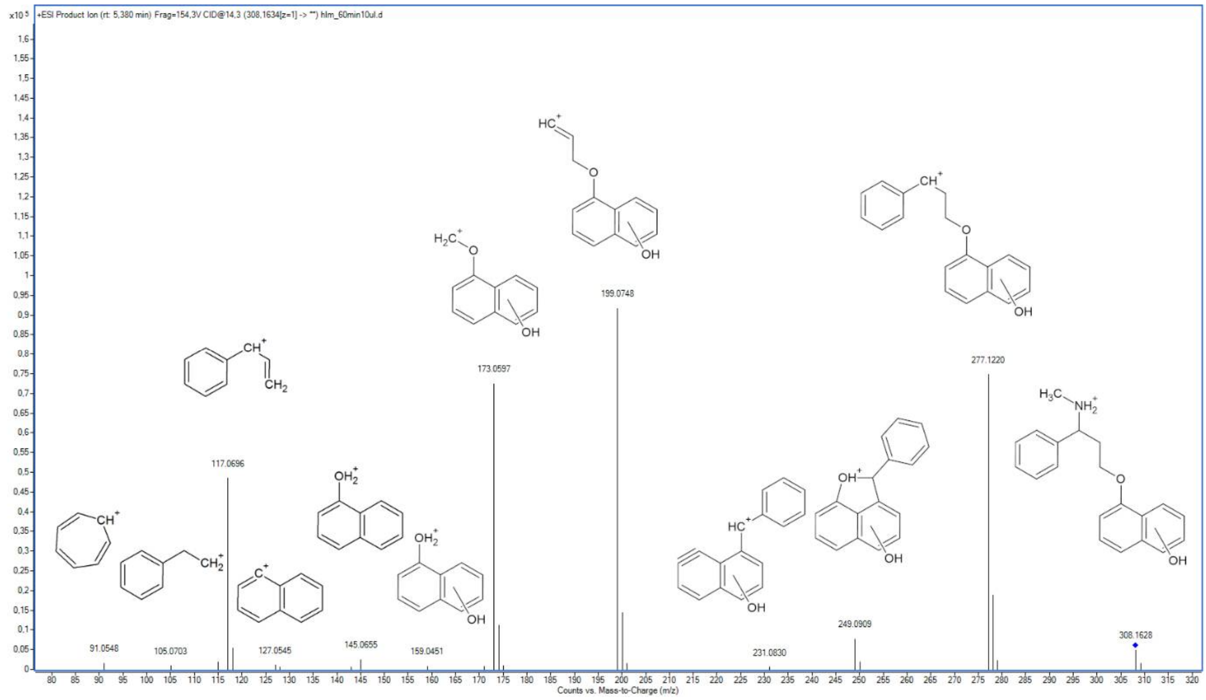

Figure S3. MS/MS spectrum and fragmentation pattern of M2

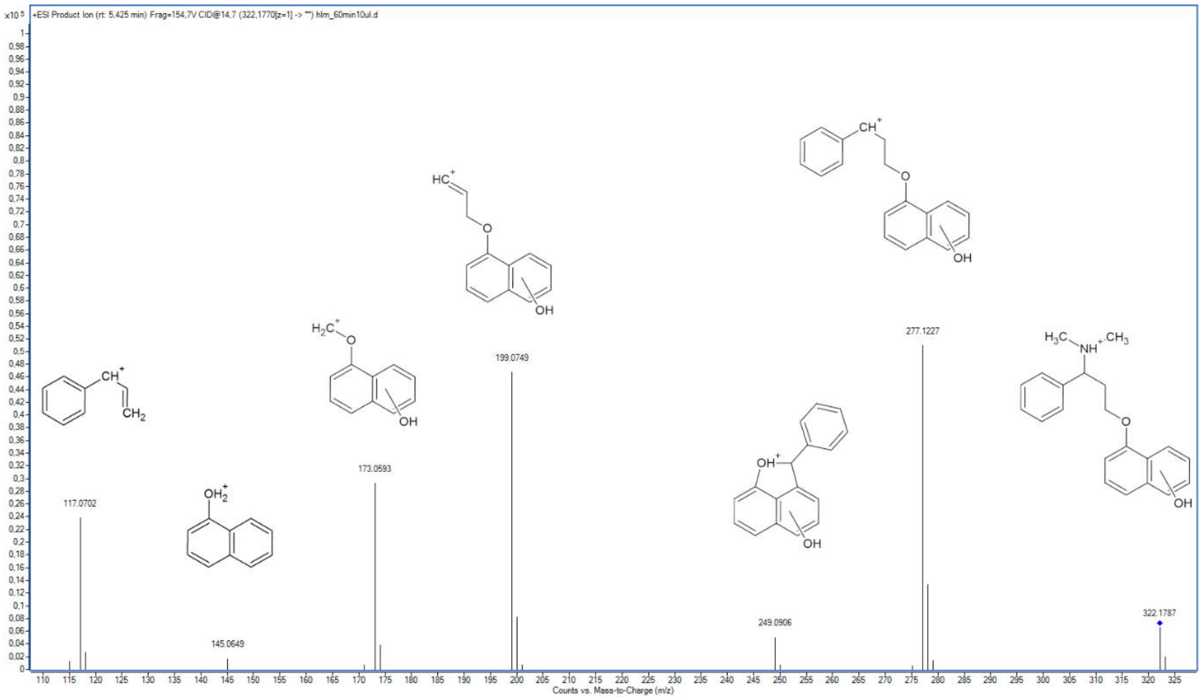

Figure S4. MS/MS spectrum and fragmentation pattern of M3

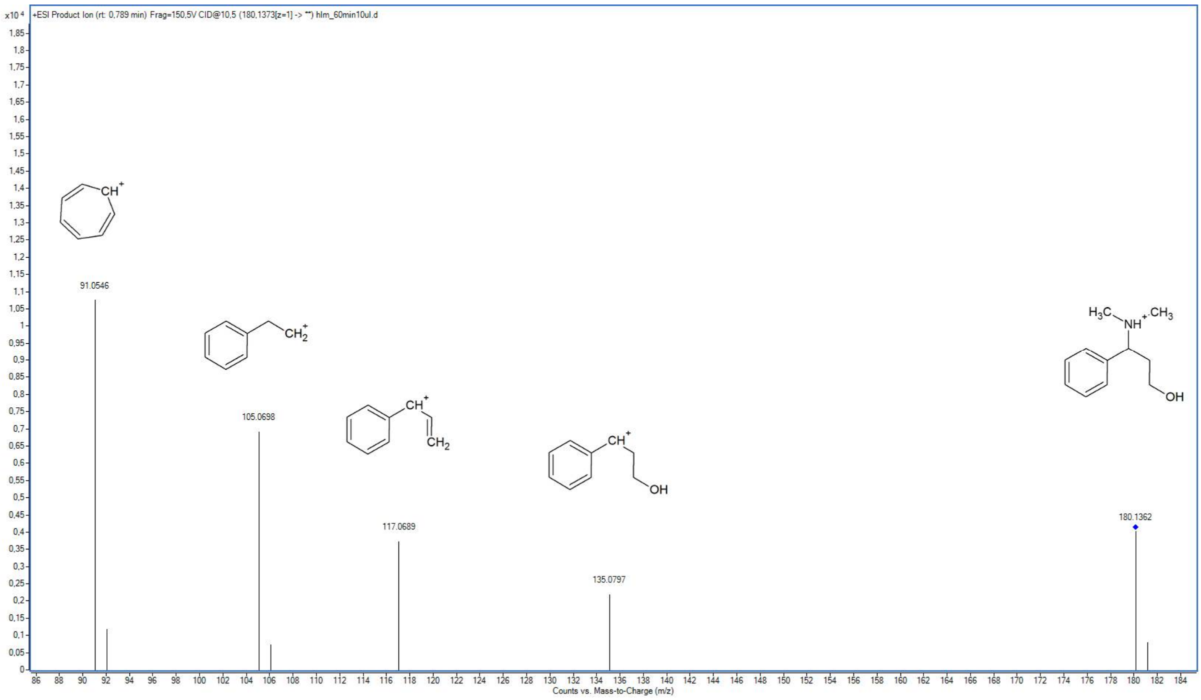

Figure S5. MS/MS spectrum and fragmentation pattern of M4

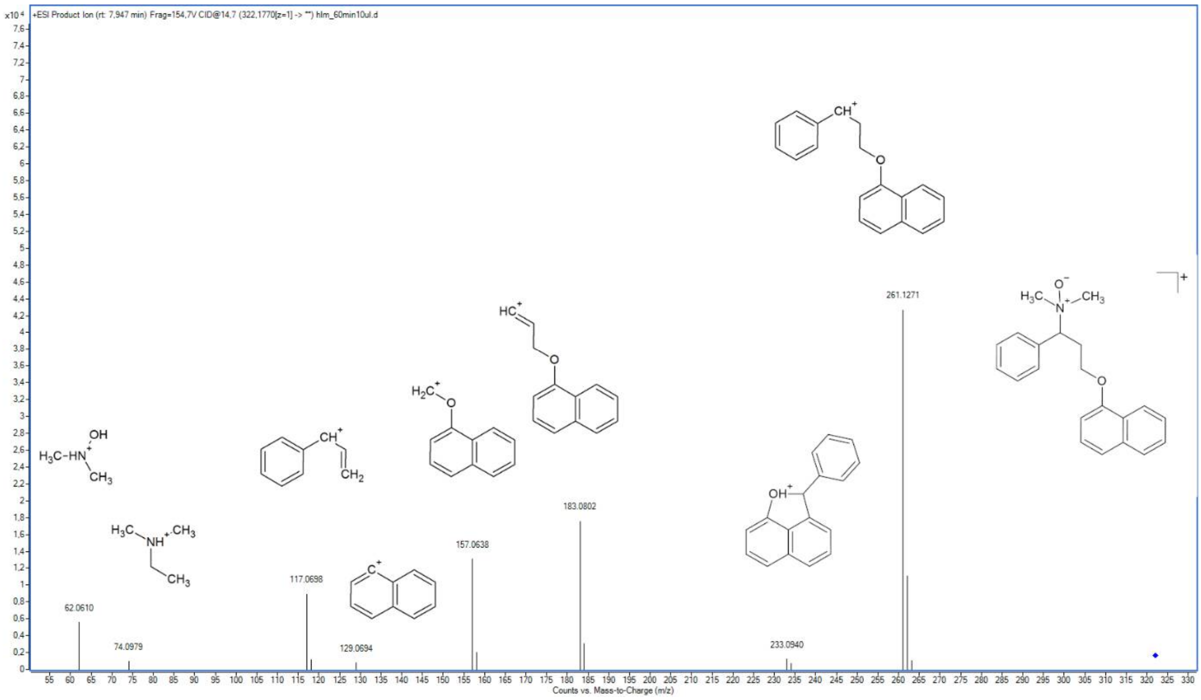

Figure S6. MS/MS spectrum and fragmentation pattern of M5

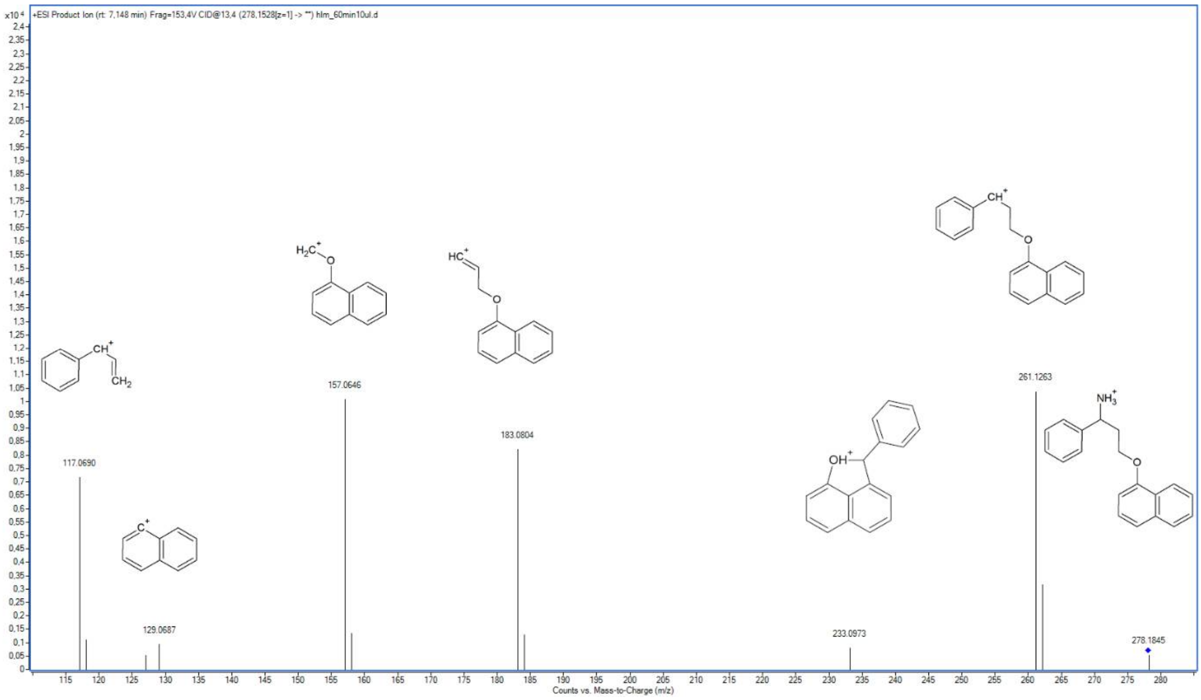

Figure S7. MS/MS spectrum and fragmentation pattern of M6

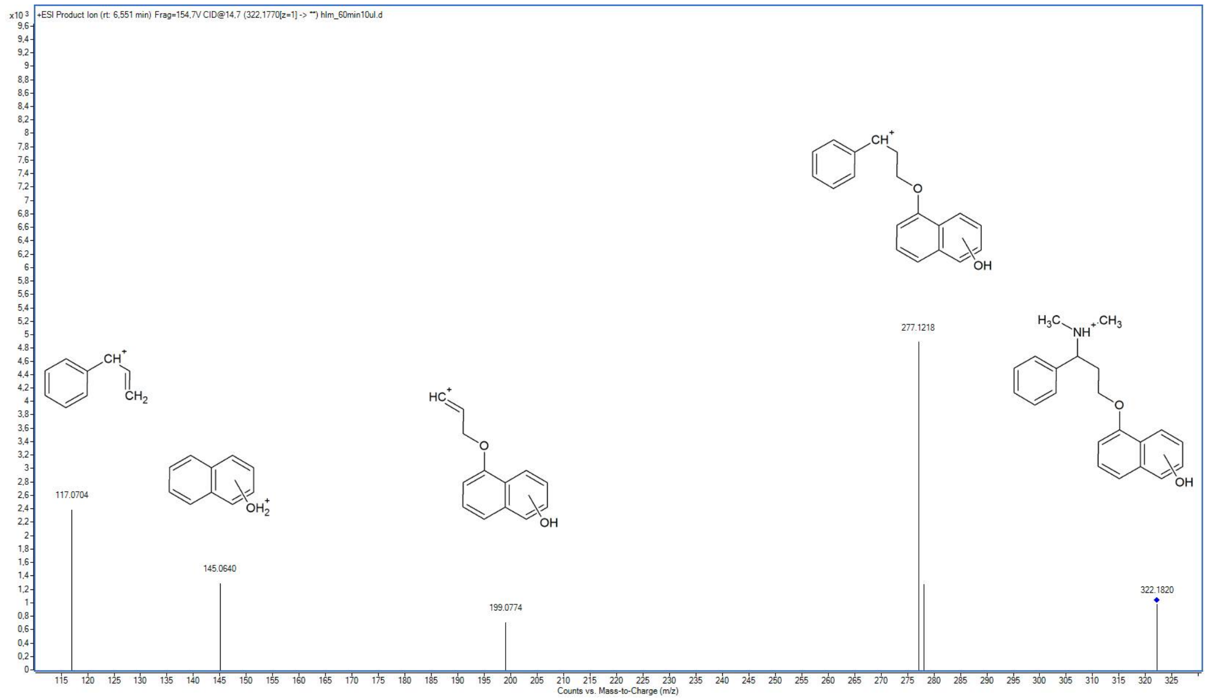

Figure S8. MS/MS spectrum and fragmentation pattern of M7

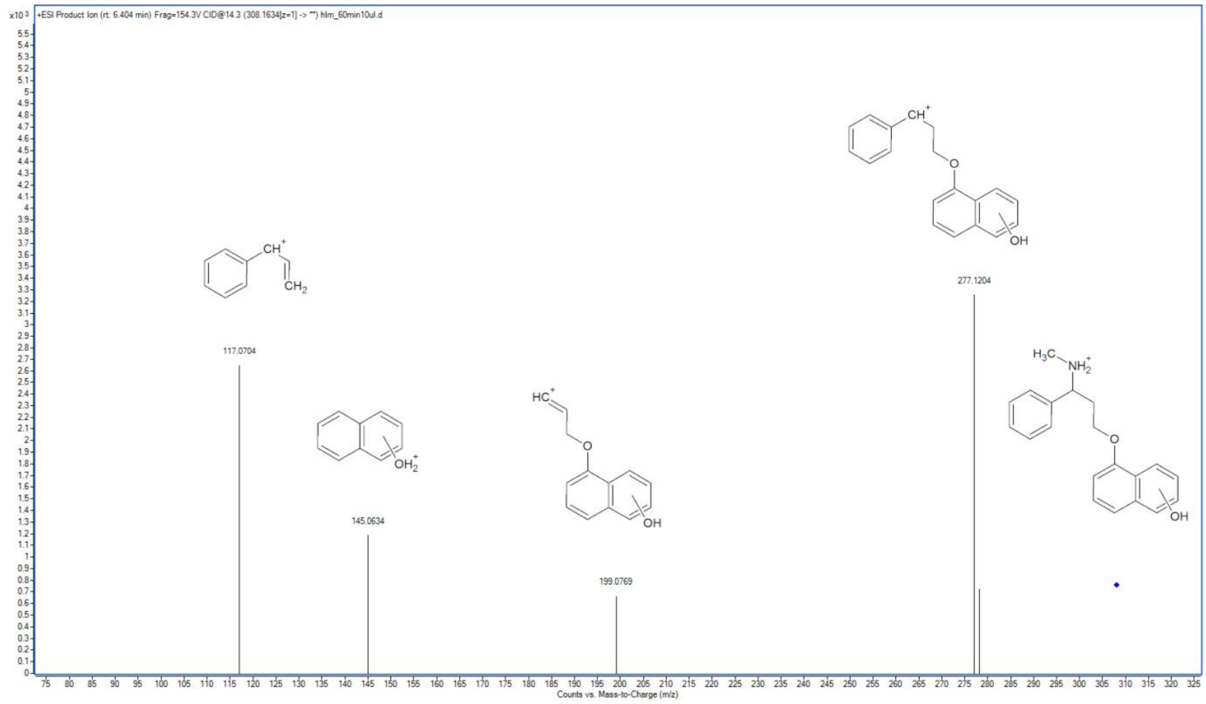

Figure S9. MS/MS spectrum and fragmentation pattern of M8

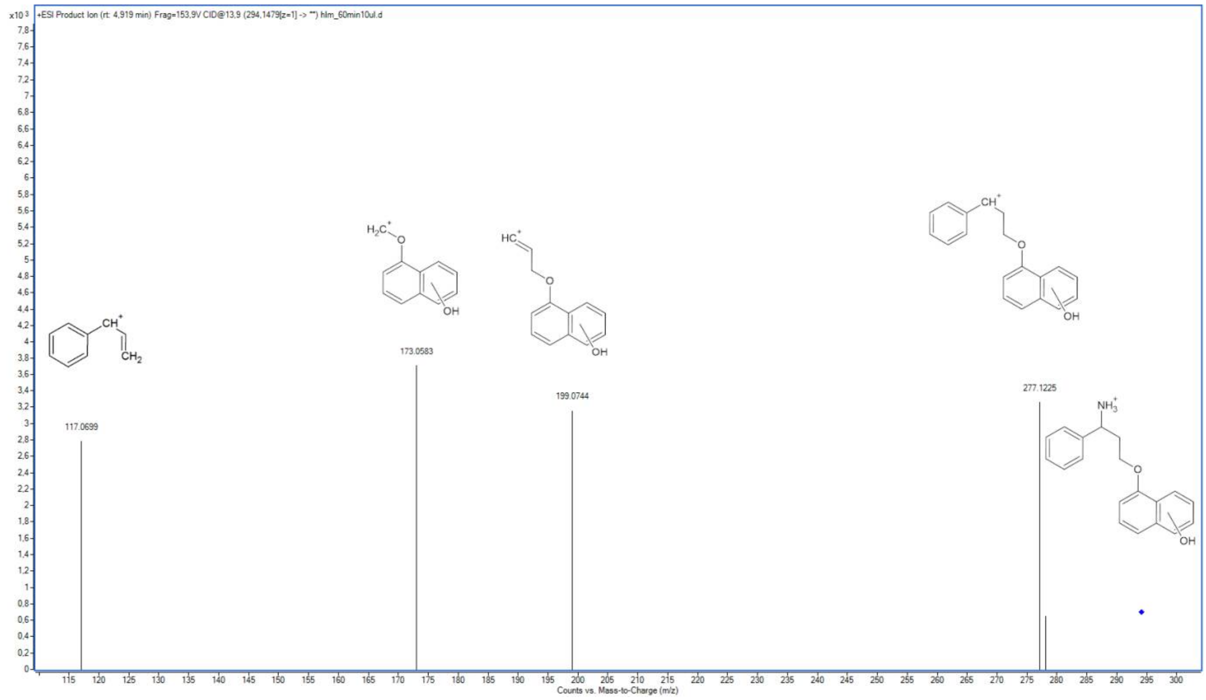

Figure S10. MS/MS spectrum and fragmentation pattern of M9

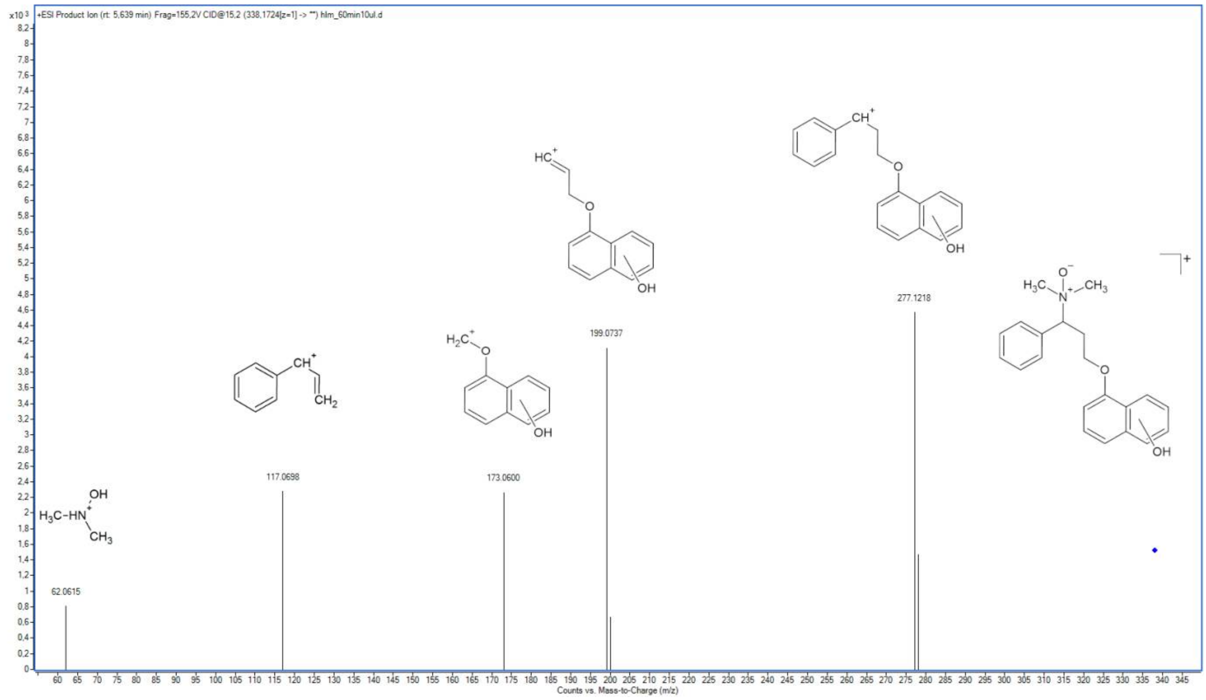

Figure S11. MS/MS spectrum and fragmentation pattern of M10

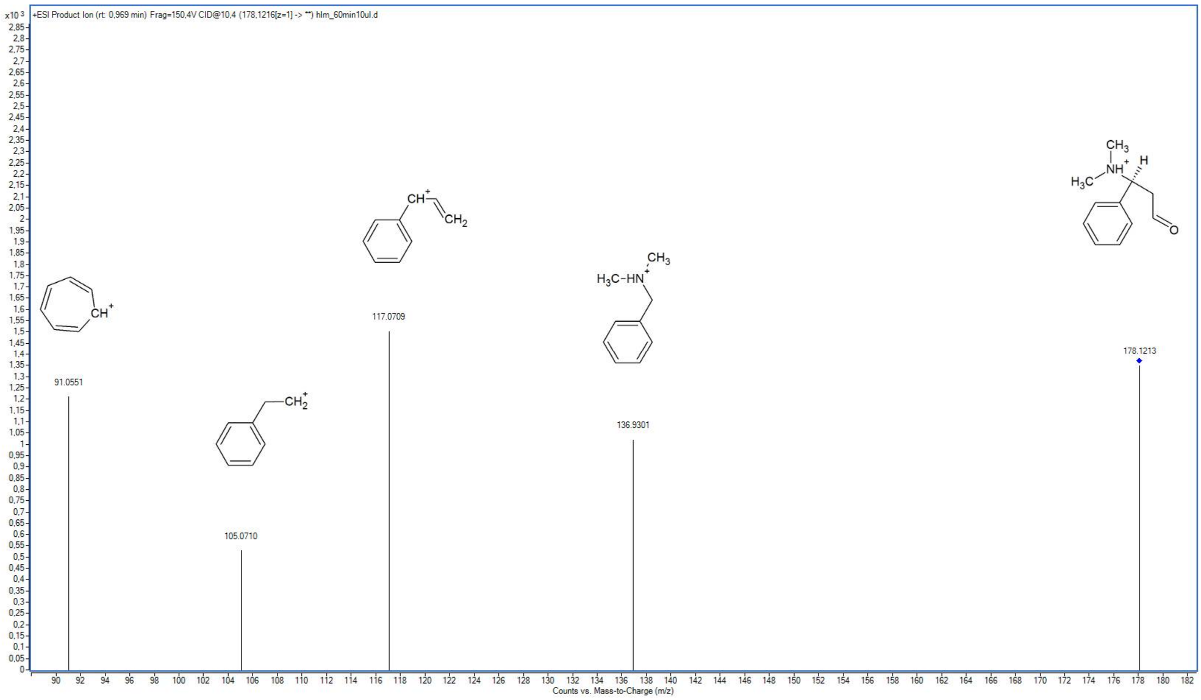

Figure S12. MS/MS spectrum and fragmentation pattern of M11
